# Menstrual Health Education Using a Specialized Large Language Model in India: Development and Evaluation Study of MenstLLaMA

**DOI:** 10.2196/71977

**Published:** 2025-07-16

**Authors:** Prottay Kumar Adhikary, Isha Motiyani, Gayatri Oke, Maithili Joshi, Kanupriya Pathak, Salam Michael Singh, Tanmoy Chakraborty

**Affiliations:** 1 Department of Electrical Engineering, Indian Institute of Technology Delhi, Room: 3B-7 (Block III 3rd Floor), Hauz Khas, New Delhi, 110016, India, 91 26591076 ext 011; 2 Yardi School of Artificial Intelligence, Indian Institute of Technology Delhi, New Delhi, India

**Keywords:** menstrual health education, artificial intelligence, large language model, cultural sensitivity, health equity, digital health

## Abstract

**Background:**

The quality and accessibility of menstrual health education (MHE) in low- and middle-income countries, including India, remain inadequate due to persistent challenges (eg, poverty, social stigma, and gender inequality). While community-driven initiatives have sought to raise awareness, artificial intelligence offers a scalable and efficient solution for disseminating accurate information. However, existing general-purpose large language models (LLMs) are often ill-suited for this task, tending to exhibit low accuracy, cultural insensitivity, and overly complex responses. To address these limitations, we developed MenstLLaMA—a specialized LLM tailored to the Indian context and designed to deliver MHE empathetically, supportively, and accessibly.

**Objective:**

We aimed to develop and evaluate MenstLLaMA—a specialized LLM tailored to deliver accurate, culturally sensitive MHE—and assess its effectiveness in comparison to existing general-purpose models.

**Methods:**

We curated MENST—a novel, domain-specific dataset comprising 23,820 question-answer pairs aggregated from medical websites, government portals, and health education resources. This dataset was systematically annotated with metadata capturing age groups, regions, topics, and sociocultural contexts. MenstLLaMA was developed by fine-tuning Meta-LLaMA-3-8B-Instruct, using parameter-efficient fine-tuning with low-rank adaptation to achieve domain alignment while minimizing computational overhead. We benchmarked MenstLLaMA against 9 state-of-the-art general-purpose LLMs, including GPT-4o, Claude-3, Gemini 1.5 Pro, and Mistral. The evaluation followed a multilayered framework: (1) automatic evaluation using standard natural language processing metrics (BLEU [Bilingual Evaluation Understudy], METEOR [Metric for Evaluation of Translation with Explicit Ordering], ROUGE-L [Recall-Oriented Understudy for Gisting Evaluation-Longest Common Subsequence], and BERTScore [Bidirectional Encoder Representations from Transformers Score]); (2) evaluation by clinical experts (N=18), who rated 200 expert-curated queries for accuracy and appropriateness; (3) medical practitioner interaction through the ISHA (Intelligent System for Menstrual Health Assistance) interactive chatbot, assessing qualitative dimensions (eg, *relevance, understandability, preciseness, correctness,* and *context sensitivity*); and (4) a user study with volunteer participants (N=200), who evaluated MenstLLaMA in 15- to 20-minute randomized sessions, rating the system across 7 qualitative user satisfaction metrics.

**Results:**

MenstLLaMA achieved the highest scores in BLEU (0.059) and BERTScore (0.911), outperforming GPT-4o (BLEU: 0.052, BERTScore: 0.896) and Claude-3 (BERTScore: 0.888). Clinical experts preferred MenstLLaMA’s responses over gold-standard answers in several culturally sensitive cases. In medical practitioners’ evaluations using the ISHA—the chat interface powered by MenstLLaMA—the model scored 3.5 in *relevance*, 3.6 in *understandability*, 3.1/5 in *preciseness*, 3.5/5 in *correctness*, and 4.0/5 in *context sensitivity*. User evaluations indicated even stronger results, with ratings of 4.7/5 for *understandability*, 4.3/5 for *relevance*, 4.28/5 for *preciseness*, 4.1/5 for *correctness*, 4.6/5 for *tone*, 4.2/5 for *flow*, and 3.9/5 for *context sensitivity*.

**Conclusions:**

MenstLLaMA demonstrates exceptional accuracy, empathy, and user satisfaction within the domain of MHE, bridging critical gaps left by general-purpose LLMs. Its potential for integration into broader health education platforms positions it as a transformative tool for menstrual well-being. Future research could explore its long-term impact on public perception and menstrual hygiene practices, while expanding demographic representation, enhancing context sensitivity, and integrating multimodal and voice-based interactions to improve accessibility across diverse user groups.

## Introduction

### Overview

Menstruation, a biological process experienced by nearly half the global population, continues to be shrouded in stigma and misinformation, particularly in low-income and rural areas [1]. This highlights the widespread lack of awareness about menstrual hygiene, resulting in health risks and perpetuating myths. In India, menstrual health remains a deeply ingrained taboo across both rural and urban settings [2]. Women are often segregated during menstruation, barred from entering places of worship, and labeled “impure” or “unclean” during this natural cycle [3,4]. This societal ostracism, coupled with inadequate menstrual health education (MHE), has far-reaching consequences. For instance, nearly 24% of school-aged girls in India skip or drop out of school altogether due to menstruation-related issues [5], reflecting the persistent discrimination they face from a young age [2]. These challenges underscore the urgent need for nationwide initiatives to make MHE accessible, inclusive, and culturally sensitive.

MHE can empower women and benefit society in multiple ways. It can provide essential information about maintaining menstrual hygiene and accessing supplies and sanitation facilities, thereby preventing menstrual health disorders [6]. Simultaneously, it can address the social stigma surrounding menstruation and help dismantle cultural taboos that restrict women from fully participating in various spheres of life while menstruating [6]. While India’s education system has begun incorporating MHE into school curricula, these efforts often fall short, failing to address cultural sensitivities or challenge the deeply rooted menstrual stigma in society [7]. Nongovernmental organizations have stepped in to bridge this gap by conducting community-based workshops and awareness campaigns nationwide [7]. However, the advent of generative artificial intelligence (AI) and large language models (LLMs) offers an unprecedented opportunity to scale these efforts and reshape public understanding of menstrual health.

Most of the Indian population, particularly the youth, has internet access and relies on it as a primary source of knowledge. However, while the internet offers a vast repository of information, it is also prone to spreading misinformation and reinforcing the stigma associated with menstruation [8]. The introduction of LLMs offers new possibilities for disseminating accurate menstrual health information and combating misinformation [9]. Compared with simpler interventions such as static mobile apps or traditional chatbots, LLMs provide dynamic, conversational, and context-aware responses that can adapt to user queries more naturally and empathetically. This adaptability is especially important for culturally sensitive topics such as menstruation, where rigid or templated responses may fall short. Moreover, a single LLM-based solution can meet diverse linguistic and contextual needs without requiring extensive hard-coded rules, making it a scalable tool even in low-resource settings where specialized infrastructure or ongoing maintenance may be limited. Although LLMs hold promise as tools for disseminating accurate health information, existing general-purpose models often fall short in addressing this specialized domain. They tend to struggle with low accuracy, a lack of cultural sensitivity, and a tendency to generate verbose or overly complex responses that fail to resonate with diverse user groups [10]. In recent times, many chatbot-based solutions have emerged, such as Flo [11] and SnehAI [12]. These chatbots have advanced MHE by providing personalized cycle tracking, symptom management, and demonstrating efficacy in improving health literacy and user engagement [13]. Despite these advancements, existing chatbots often face limitations. They tend to offer shallow conversational interactions, limited flexibility, and difficulty adapting to diverse cultural backgrounds. Additionally, their primary focus remains on symptom monitoring and cycle prediction rather than offering a more comprehensive and empathetic educational experience. These gaps highlight the need for specialized, culturally adaptive language models capable of delivering thorough MHE in an interactive and inclusive manner.

To tackle these challenges, this study aims to develop and evaluate MenstLLaMA, a specialized LLM tailored for MHE, and to assess its effectiveness in terms of clinical accuracy, cultural sensitivity, and user satisfaction, in comparison to existing general-purpose LLMs. Built upon the LLaMA-3-8B-Instruct model [14] and fine-tuned using our custom MENST dataset comprising 23,820 question-answer (QA) pairs, MenstLLaMA addresses critical gaps in current LLM capabilities. Our comprehensive evaluation demonstrated MenstLLaMA’s superior performance compared with leading models, including GPT-4o [15] and Claude-3 [16], based on both automated metrics and clinical expert assessments. To facilitate evaluation by medical practitioners and to conduct a user study, we developed ISHA (Intelligent System for Menstrual Health Assistance), an interactive chatbot powered by MenstLLaMA. This study highlights the potential of MenstLLaMA as an empathetic, culturally aware AI companion for advancing MHE in India.

### Related Work

LLMs have shown promise across various health care domains; however, their application to MHE remains limited. Before initiating this study, we conducted a systematic review of AI applications in MHE, searching PubMed, Google Scholar, and arXiv for articles published between 2020 and 2024 using the keywords “artificial intelligence,” “language models,” “menstrual health,” and “health education.” While general-purpose LLMs such as GPT-4 (OpenAI, Inc) and Claude-3 (Anthropic PBC) have demonstrated effectiveness in medical knowledge tasks, no specialized models currently exist for MHE. Moreover, these general-purpose models are prone to hallucinations, that is, generating fluent but factually incorrect medical content [17]. This poses a serious risk to the safety and trustworthiness of health care apps [18], particularly in sensitive domains such as menstrual health, where inaccurate advice can result in misinformation and potential harm. Previous AI efforts in this domain have primarily focused on period tracking and symptom prediction, with limited emphasis on providing comprehensive MHE or counseling through specialized LLMs. Delivering accurate and accessible menstrual health information at scale remains a major challenge for health care systems globally. Resource limitations and societal barriers constrain traditional approaches that rely on health care professionals and formal educational programs. Recent advances in AI and natural language processing offer promising avenues for delivering personalized MHE. Nonetheless, general-purpose LLMs often struggle with cultural insensitivity, producing responses that misalign with local norms and sociocultural taboos surrounding menstruation [19]. These limitations hinder effective communication in culturally nuanced contexts, highlighting the need for domain-specific, culturally aware models. However, research on specialized language models for sensitive health care domains remains limited, especially in culturally nuanced areas such as menstrual health. Most existing studies in this domain focus on chatbot-based interventions. For instance, Cunningham et al [13] used Flo [11], a period and reproductive health tracker that offers personalized menstruation and ovulation predictions, symptom forecasts, expert-reviewed content, and an anonymous community platform. They used the app to assess its efficacy in improving menstrual health literacy and well-being outcomes, in both individuals with and without premenstrual syndrome and premenstrual dysphoric disorder. Kim et al [20] conducted a randomized controlled trial demonstrating that co-designed mobile educational modules significantly improved sexual knowledge (video format) and menstrual knowledge (PDF), with comparable overall effectiveness between the 2 formats. Another study reported the development of an AI-based reproductive and menstrual health learning module, which was found to be effective as an educational tool. It addressed cognitive accessibility through revised materials and interactive design [21], and its feasibility was validated by students, teachers, and experts. While mobile apps for menstrual tracking have shown promise in facilitating real-time monitoring of menstrual health, recent studies indicate that app users and nontrackers share comparable demographic and menstrual cycle characteristics, suggesting the potential broad applicability of digital interventions in this domain [22]. This is further supported by chatbots such as SnehAI, which has effectively addressed sensitive health topics in India by engaging millions of users in discussions about taboo subjects while delivering accurate and culturally appropriate information [12]. Beyond chatbots, research also shows that web-based menstrual health resources can improve health literacy and encourage medical consultation among young people [23]. However, these platforms generally offer limited interactivity.

Additionally, educational interventions have shown promise in improving menstrual health knowledge. For instance, a study in Iran demonstrated that structured health education programs significantly improved menstrual health knowledge among adolescent girls, highlighting the effectiveness of targeted educational approaches [24]. Sosnowski et al [25] proposed a hierarchical 3-layer architecture that analyzes menstrual cycle features to predict ovulation dates and detect health risks such as premenstrual syndrome and luteal phase defect. Although this approach showed success in cycle prediction and risk assessment, its focus remained primarily on physiological tracking rather than comprehensive MHE.

## Methods

### Study Design and Setting

This study followed a structured approach, encompassing 3 main stages: dataset creation, model development, and evaluation. In the first stage, we developed the MENST dataset, a comprehensive collection of 23,820 QA pairs covering various facets of menstrual health. This dataset served as the foundation for the development and evaluation of MenstLLaMA, a novel generative AI model built upon the Large Language Model Meta AI 3 (LLaMA-3) architecture [11].

In the model development phase, we fine-tuned the LLaMA-3 model using the MENST dataset to enhance its ability to generate accurate, culturally sensitive, and empathetic responses for MHE. To evaluate MenstLLaMA’s performance, we conducted a comparative analysis with other state-of-the-art GenAI models (eg, GPT-4o and Claude-3) and assessed its effectiveness through expert clinical assessments.

Model fine-tuning and analysis were performed using Python-based machine learning frameworks (Python Foundation), including PyTorch and Hugging Face’s Transformers library [26]. The fine-tuning process leveraged high-performance computing resources to optimize model performance and efficiency. For comprehensive statistical analysis, we used the SciPy [27] and NLTK [28] libraries, calculating various evaluation metrics to ensure reproducibility and robustness in our assessments. In addition to these computational evaluations, human-based assessments were conducted by clinical experts and end users. These evaluations focused on the model’s relevance, understandability, and cultural sensitivity, particularly in the context of MHE. This dual approach ensured a thorough and well-rounded evaluation process.

### Dataset Creation

#### Dataset Sources

The MENST dataset was compiled from a range of reputable sources, including health information portals, medical institutions, government websites, global organizations, and educational platforms. Most datasets were sourced from official medical documents, from which we extracted and curated relevant frequently asked questions (FAQs). Additionally, we incorporated an existing QA dataset on menstrual health, specifically the Menstrual Health Awareness Dataset [29], which includes 1986 QA pairs. All collected QA pairs, including those from external sources, were manually annotated with metadata (see the “Metadata Creation” section for a detailed description).

To further enrich the dataset, we used prompting techniques using advanced language models such as GPT-4 and Gemini 1.5 Pro to generate additional QA pairs based on relevant menstrual health documents. This approach contributed to the development of a robust and comprehensive dataset tailored for MHE, with a strong focus on accuracy, cultural relevance, and empathetic tone. In the following subsections, we provide a detailed breakdown of the dataset’s structure, metadata creation, and the paraphrasing strategies used to augment its coverage.

#### Metadata Creation

We created comprehensive metadata for all documents to facilitate efficient data management and detailed cataloging of menstrual health topics across various demographics and contexts. Each document was tagged with a unique document ID, beginning with either “D” or “F”: unstructured documents or paragraphs were prefixed with “D,” denoting “documents,” while structured documents in the form of FAQs were prefixed with “F.” The metadata also included the source and URL of each document, along with the menstrual health topics covered.

As fine-tuning an LLM for tasks such as QA or conversational interaction requires a QA format, we transformed unstructured documents into QA pairs using LLMs. The preprocessing methodology for this transformation is described in detail in the following section. These LLM-generated QA pairs were then combined with preexisting pairs to construct the final MENST dataset. Table 1 presents the metadata elements for the original collected documents, while Table 2 outlines the metadata schema for the final processed QA dataset.

**Figure 1.**
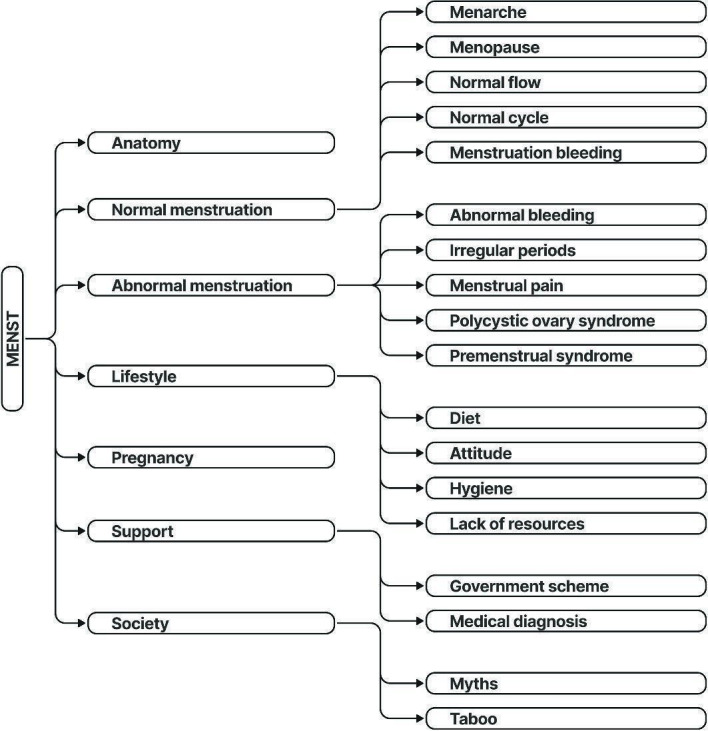
Taxonomy of menstrual health topics in the MENST dataset. The taxonomy consists of 7 primary categories (anatomy, normal menstruation, abnormal menstruation, pregnancy, lifestyle, support, and society) with corresponding subtopics as described in the *Metadata Creation* section.

In collaboration with expert gynecologists, we developed a taxonomy to categorize the topics covered in the menstrual health documents. The taxonomy consists of primary categories including *anatomy*, *normal menstruation*, *abnormal menstruation*, *pregnancy*, *lifestyle*, *support*, and *society*. Each primary category is further subdivided into specific subtopics for greater thematic precision. For instance, the *normal menstruation* category includes subtopics such as *menarche*, *menopause*, *normal flow*, and *normal cycle*, while the *abnormal menstruation* category covers issues such as *abnormal bleeding*, *irregular periods*, *menstrual pain*, *polycystic ovary syndrome*, and *premenstrual syndrome*. A detailed breakdown of the dataset’s taxonomy is provided in Figure 1.

**Table 1.** Metadata schema for source documents in the MENST dataset.

Metadata labels	Description
Document ID	Each document is assigned a unique ID, starting with “D” or “F.” This naming convention helps distinguish documents with only paragraph texts and question answers.
Document Name	Name of the document, such as title or main heading.
Source	Source of the document containing the name of the website or organization.
Link	URL taking to the document
Topic	The topic on which the document is based, for example, irregular menstruation.

**Table 2.** Metadata schema for question-answer pairs in the MENST dataset.

Metadata labels	Description
Question	Questions related to the topic.
Answer	Answer to the question.
Age group	Age group to which the question is related. Different age groups are considered, including adolescents (those below 19 years), young adults (those between 20 and 40 years), and older adults (those above 40 years).
Region	The region the question is specific about. It could be rural, urban, or both.
Topic	The topic on which the question is based.
Subtopic	This specifies the exact subdomain of the question under the topic.
Keywords	Tags contained in the query (ie, medication, therapy, surgery, or doctor).
Document ID	ID of the document from which the question has been created or taken.
LLM^a^ Used	Name of the LLM used for postprocessing the question.

a LLM: large language model.

#### Question-Answer Pair Creation

Our primary dataset consisted of 88 documents, of which 13 were structured as QA (FAQ) documents. These documents, sourced from official medical portals, were designated as a gold test set (Set-1). The remaining 75 unstructured documents were processed using GPT-4 and Gemini 1.5 Pro to generate QA pairs, which were subsequently validated by domain experts.

**Figure 2.**
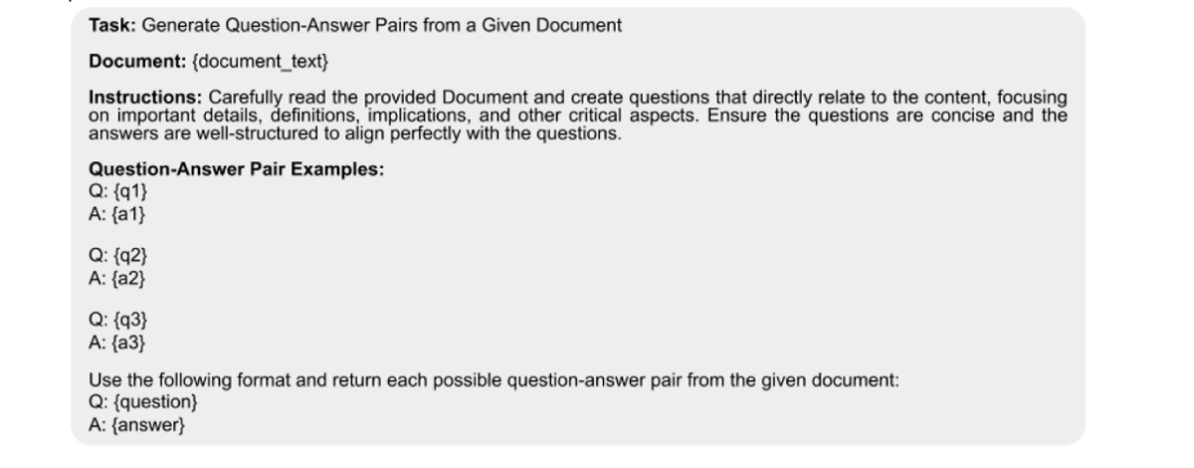
Prompt template structure used for curating question-answer pairs from unstructured medical documents. The template includes 4 components: (1) a task description specifying the objective of generating relevant questions from the document content; (2) the unstructured document text from which the question-answer (QA) pair is to be generated; (3) instructions for formulating concise, well-structured questions focusing on key information; and (4) example QA pairs (n=3) from gold-standard data to guide the generation process.

To ensure consistency in the QA generation process, we used a standardized prompt template. This prompt template included 3 randomly selected QA pairs from Set-1 to provide contextual examples for the language models. The prompt format is illustrated in Figure 2.

#### Paraphrasing for Dataset Augmentation

**Figure 3.**
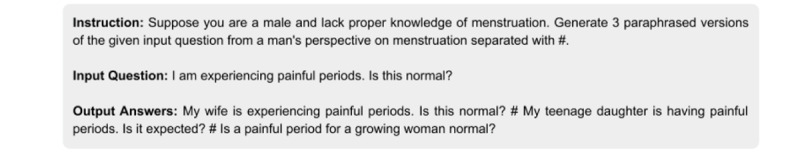
Example of the prompt template used for dataset augmentation through question paraphrasing. The template illustrates the male-perspective paraphrasing strategy, comprising (1) an input instruction specifying the task of rephrasing a question from a male viewpoint; (2) a sample input question related to menstrual pain; and (3) 3 generated paraphrased outputs reflecting different female familial roles (wife, teenage daughter, and growing woman). This strategy enabled the generation of diverse yet contextually relevant question variants, enhancing MenstLLaMA’s ability to understand and respond to queries from varied social and cultural perspectives. LLaMA: Large Language Model Meta AI.

To enhance the diversity of the dataset, we applied paraphrasing techniques, aiming to improve the model’s ability to handle varied user expressions and contextual nuances. By prompting GPT-3.5 with specific instructions, as shown in Figure 3, we implemented 4 distinct paraphrasing strategies.

- Scenario-based paraphrasing introduces real-life contexts into the questions, making the training data more relatable and reflective of natural human interactions. For example, the question “Can I cook while I am on my period?” can be paraphrased as “Today while I was in the kitchen, my mother scolded me as I entered the kitchen on my period. Is it a sin to cook during this phase?” This approach helps the model interpret questions more effectively and respond in ways that align with human conversational patterns.
- Rephrasing from a male point of view provides a perspective often lacking direct experience with menstruation. This viewpoint is essential, as it expands the model’s understanding and enables it to engage with individuals indirectly affected by menstrual health. For example, “I am experiencing painful periods. Is this normal?” can be rephrased as “My wife is experiencing painful periods. Is this normal?” This ensures the model can adequately address queries from a wider audience.
- Changing the sentence structure introduces linguistic diversity, which is crucial for developing syntactic flexibility in a model. For example, “Is it normal to start menstruating at the age of 12?” can be rephrased as “I am 12 years old and just got my first period. Is this okay?” Such variations help the model understand and respond to differently phrased but semantically similar questions.
- Rephrasing from a male point of view provides a perspective often lacking direct experience with menstruation. This viewpoint is essential, as it expands the model’s understanding and enables it to engage with individuals indirectly affected by menstrual health. For example, “I am experiencing painful periods. Is this normal?” can be rephrased as “My wife is experiencing painful periods. Is this normal?” This ensures the model can adequately address queries from a wider audience.
- Changing the sentence structure introduces linguistic diversity, which is crucial for developing syntactic flexibility in a model. For example, “Is it normal to start menstruating at the age of 12?” can be rephrased as “I am 12 years old and just got my first period. Is this okay?” Such variations help the model understand and respond to differently phrased but semantically similar questions.
- Paraphrasing from the perspective of rural women captures the language and concerns of individuals from rural settings, who may have distinct cultural and educational backgrounds. For example, “I don’t have access to sanitary pads. What can I use instead?” can be paraphrased as “In my village, there are no sanitary pads. What can I use instead?” This helps the model provide contextually appropriate responses for a diverse demographic, especially for users from underserved communities such as rural students.

Each question from the raw dataset was paraphrased using 4 distinct strategies, resulting in 23,820 QA pairs. A detailed breakdown of the dataset’s structure is provided in Table 3.

**Table 3.** Distribution of question-answer pairs in the MENST dataset across different data sources before and after paraphrasing augmentation.

Set no.	Source	Raw question-answer pairs (N=1985), n	Paraphrased question-answer pairs (N=23,820), n
Set-1	Frequently asked questions	94	1128
Set-2	Text documents on menstruation	1319	15,828
Set-3	Menstrual health awareness dataset	572	6864

#### Expert-Compiled Test Dataset

In collaborating with medical professionals, we developed an expert-curated test dataset to evaluate the model’s performance. Experts were asked to compile questions they commonly encounter in clinical practice and to provide corresponding answers based solely on their clinical expertise, without consulting external materials. Using our metadata framework (7 topics and 19 subtopics), we compiled 200 QA pairs reflecting real-life clinical scenarios. This expert-curated dataset served as a gold test set for benchmarking the model’s performance.

### Model Development

#### Overview

We developed MenstLLaMA, a specialized language model for menstrual health, by fine-tuning the Meta-LLaMA-3-8B-Instruct model [14] on our curated MENST dataset, which comprises 23,820 QA pairs. Fine-tuning was performed using a parameter-efficient fine-tuning (PEFT) strategy [30], with a specific focus on low-rank adaptation (LoRA) [31] to align the model with the MENST dataset. PEFT is an adapter-based fine-tuning approach that optimizes training efficiency by updating only a small subset of model parameters, while keeping the majority of the base model frozen. LoRA, in particular, inserts low-rank matrices into the attention mechanism, enabling the model to learn task-specific patterns effectively with reduced computational overhead. We also considered alternative strategies, including full fine-tuning, other adapter-based methods (eg, prefix-tuning, prompt-tuning), and retrieval-augmented generation. However, full fine-tuning is resource-intensive and less practical for iterative experimentation. Retrieval-augmented generation, while powerful, introduces a dependency on external retrieval systems that may not always yield culturally sensitive or domain-relevant content. Although other adapter-based methods are lightweight, LoRA provides the most favorable trade-off between computational efficiency, performance, and ease of integration with transformer-based architectures. Given these advantages, LoRA was selected as the most suitable approach for domain-adaptive fine-tuning of MenstLLaMA. This method allows for efficient adaptation across diverse conversational contexts while maintaining performance comparable to full fine-tuning.

**Figure 4.**
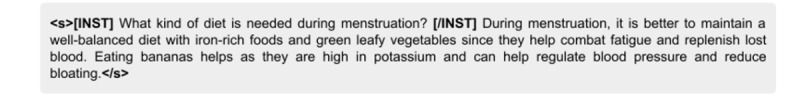
Instruction fine-tuning format used for MenstLLaMA, illustrating the standardized structure for question-answer (QA) pairs. The format shows the conversion of QA pairs into LLaMA instruction syntax (<s > [INST] question [/INST] answer<s >). This example features a dietary question related to menstruation, with a response providing clear, evidence-based nutritional guidance. This format helps the model effectively distinguish between questions and their corresponding answers. LLaMA: Large Language Model Meta AI.

Instruction fine-tuning was performed on the LLaMA-3-8B-Instruct model using a structured prompt format designed to distinguish between instructions and responses. Each QA pair was reformatted accordingly, as illustrated in Figure 4. This structure helped the model accurately identify and interpret, enhancing its ability to manage complex, context-sensitive conversations. By explicitly delineating instructions from responses, the model was better equipped to capture the nuances of dialogue and generate more coherent, contextually appropriate outputs.

We used Meta-Llama-3-8B-instruct [32], an 8-billion parameter model, as the base for our configuration. Fine-tuning was performed with a maximum sequence length of 2048 tokens, utilizing LoRA for parameter-efficient training. To optimize memory usage, we applied 4-bit quantization using the NF4 data type. The model was trained using the Paged AdamW [33] optimizer with 32-bit precision to ensure efficient gradient updates. Training hyperparameters were carefully fine-tuned to balance learning rate, batch size, and gradient accumulation steps, achieving optimal performance with minimal computational overhead. The fine-tuning process was conducted over 5 epochs, with a learning rate of 2 × 10^–4^, a warmup ratio of 0.03, and a maximum gradient norm of 0.3. We used an NVIDIA A100 GPU with 80 GB of memory for model training. Notably, we leveraged free-tier API credits provided by OpenAI and Google through their academic access programs, resulting in zero financial cost for this phase. The entire process of dataset curation, prompt engineering, and API-based generation spanned approximately 10 weeks. As both fine-tuning (model training) and model deployment during the user evaluation phase were performed on local hardware (NVIDIA A100 GPU), there were no associated cloud infrastructure or compute rental costs. As such, the overall development and deployment process incurred no additional financial expenditure.

#### Development of an Interactive Chatbot

**Figure 5.**
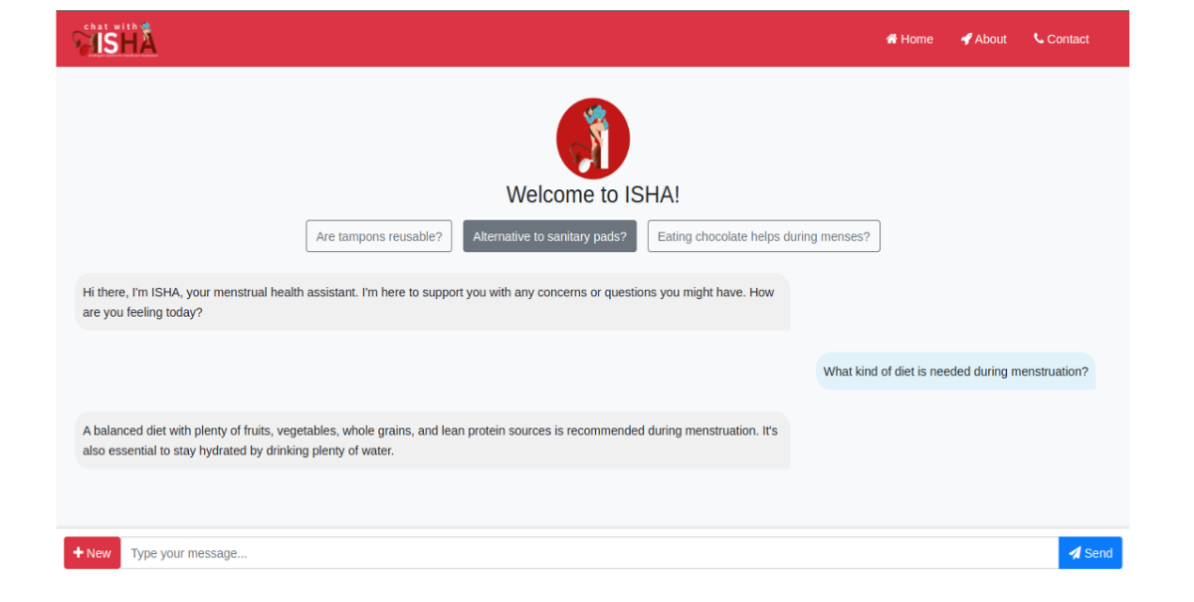
Interface of the ISHA (Intelligent System for Menstrual Health Assistance) chatbot powered by MenstLLaMA. LLaMA: Large Language Model Meta AI.

To enable qualitative evaluations by medical practitioners and facilitate case studies, we developed an interactive chatbot powered by the fine-tuned MenstLLaMA model, named ISHA. ISHA was designed to maintain seamless conversational flow by leveraging contextual information from previous utterances within the same session. During both the fine-tuning and inference stages, we incorporated tailored instructions into the model’s prompts to enhance the chatbot’s empathy and emotional awareness. This approach aimed to create a chatbot that is not only contextually accurate but also emotionally attuned to the nuances of the menstrual health domain. All interactions with ISHA were conducted in a controlled research environment without logging any personal identifiers, and strict data handling protocols were followed to ensure user privacy and security. Although the ISHA interface is not currently hosted for public access due to infrastructure and maintenance costs, the underlying MenstLLaMA model and supporting codebase have been made publicly available. Figure 5 presents a screenshot of the ISHA interface.

### Baseline Models

We compared MenstLLaMA against 9 state-of-the-art LLMs, including 3 closed-source models (GPT-4 [12], Gemini 1.5 Pro [34], and Claude-3 [13]) and 6 open-source models (Mistral [35], LLaMA-3 [11], GPT-2 [36], Orca 2 [37], Falcon [38], and Phi3 [39]).

### Evaluation

#### Comprehensive Evaluation Framework for MenstLLaMA and Baseline Models

The evaluation of MenstLLaMA and other state-of-the-art closed and open-sourced models followed a comprehensive 4-step approach to assess the model’s performance across technical accuracy, clinical relevance, and user satisfaction. The evaluation included *automatic evaluation* using standard natural language processing metrics, *clinical expert evaluation* on medical accuracy and appropriateness, *medical practitioner feedback* in a simulated clinical setting, and a *user-centered evaluation* for assessing accessibility and empathy. This multifaceted approach enabled a balanced and in-depth understanding of each model’s strengths and limitations, particularly in the sensitive domain of menstrual health care.

#### Automatic Evaluation

MenstLLaMA was benchmarked against both closed-source (GPT-4o, Gemini 1.5 Pro, and Claude-3) and open-source (Mistral, LLaMA-3, GPT-2, Orca 2, Falcon, and Phi3) conversational models. Given the generative nature of the task, we used standard natural language processing evaluation metrics, such as BLEU (Bilingual Evaluation Understudy) [40], METEOR (Metric for Evaluation of Translation with Explicit Ordering) [41], ROUGE-L (Recall-Oriented Understudy for Gisting Evaluation-Longest Common Subsequence) [42], and BERTScore (Bidirectional Encoder Representations from Transformers Score) [43], to assess the model outputs against the expert-compiled gold test set, evaluating lexical overlap, fluency, semantic similarity, and factual consistency. While BLEU is known to have limitations in capturing factual alignment and dialogue coherence, we included it for comparability with prior work. To address these limitations, we complemented it with BERTScore, which better captures semantic nuance and information preservation. While BLEU, METEOR, and ROUGE-L provide insights into word and sequence overlap, they are more appropriate for rigid, template-based responses. By contrast, health care dialogue often includes semantically valid variations that may not exhibit lexical similarity. In this context, BERTScore offers a more robust evaluation by capturing the semantic similarity between model outputs and expert references. Therefore, BERTScore is particularly well-suited for our domain, and we assign it greater interpretive weight when comparing model performance. Collectively, these metrics assess the fluency, relevance, overlap, and semantic similarity of the reference responses. Further details on these evaluation metrics are provided in Table 4. Based on the results of this automatic evaluation, the top 4 models were selected for further analysis. Detailed findings from this comparative evaluation are presented in the “Results” section.

**Table 4.** Evaluation metrics, definition, and scoring criteria for MenstLLaMA^a^ performance.^b^

Metric	Definition
BLEU^c^^,^^d^	Measures the precision of word overlap between the model’s response and gold reference answers, providing insight into response accuracy.
METEOR^c^^,^^e^	Evaluates semantic similarity by considering word stems, synonyms, and paraphrases, offering a more flexible assessment of meaning preservation.
ROUGE-L^c^^,^^f^	Measures the longest co-occurring subsequences between the generated response and the gold answer.
BERTScore^c^^,^^g^	Leverages contextual embeddings to gauge the semantic coherence between the gold and the generated response.
Relevance^h^^,^^i^	How closely the responses aligned with the information users sought, with ratings ranging from 1 (least relevant) to 5 (most relevant).
Understandability^h^^,^^i^	Measures the clarity and ease with which the responses could be comprehended, ensuring that the information was presented in a way that was straightforward and accessible; rated from 1 (not at all understandable) to 5 (completely understandable).
Preciseness^h^^,^^i^	The extent to which responses were concise and directly addressed the query, avoiding unnecessary details or digressions; rated from 1 (responses were random) to 5 (responses were precise).
Correctness^h^^,^^i^	The factual accuracy of the information provided; rated from 1 (not at all correct) to 5 (absolutely correct).
Context sensitivity^h^^,^^i^	The model’s ability to recognize and respond appropriately to the emotional tone of the interaction; rated from 1 (robotic) to 5 (human-like).
Tone^i^	Assesses whether the responses were delivered in a polite and empathetic manner; rated from 1 (not at all empathetic) to 5 (empathetic).
Flow^i^	States that the flow of the conversation was smooth or hard to relate to; rated from 1 (hard to relate) to 5 (smooth flow).

a LLaMA: Large Language Model Meta AI.

b For automatic evaluation metrics, all the metrics’ scores range from 0 to 1, with 0 being the least and 1 being the maximum.

c Automatic evaluation metric.

d BLEU: Bilingual Evaluation Understudy.

e METEOR: Metric for Evaluation of Translation with Explicit Ordering.

f ROUGE-L: Recall-Oriented Understudy for Gisting Evaluation-Longest Common Subsequence.

g BERTScore: Bidirectional Encoder Representations from Transformers Score.

h Medical practitioners’ assessment criteria.

i User evaluation criteria.

#### Evaluation by Clinical Experts

To assess the clinical relevance of our model against the top-performing baseline models based on automatic evaluation, we engaged 6 clinical professionals with direct experience in gynecological and sexual health consultations. The evaluation panel included 3 final-year Bachelor of Medicine trainees undergoing clinical rotations in obstetrics and gynecology, 2 certified physician assistants with a minimum of 3 years’ experience in reproductive health, and 1 licensed sexual health therapist specializing in adolescent and women’s health education. These evaluators were recruited through professional networks and academic affiliations and voluntarily participated in the study without monetary compensation. Each professional was presented with 200 questions from the expert-compiled test set. For each question, they reviewed 5 unlabeled responses: 4 generated by the top-performing models and 1 gold-standard answer curated by domain experts. To ensure objectivity, the sources of the responses were not disclosed. Evaluators were instructed to select the most clinically appropriate and clearest response for each question, based solely on content quality and relevance.

#### Evaluation by Medical Practitioners

Following the model comparisons, we further extended the evaluation by asking practicing gynecological professionals to assess MenstLLaMA’s responses in a simulated clinical setting. Using the ISHA chatbot interface, each evaluator was asked to simulate patient interactions and then rate MenstLLaMA’s responses using a set of qualitative metrics. Twelve medical practitioners were recruited: 5 experienced doctors and 7 postgraduate trainees in medicine, all of whom regularly interact with patients in their clinical routines. Recruitment for the study took place through informal, word-of-mouth referrals within health care networks. We relied on personal and professional connections to share information about the study, which helped us reach potential participants in a more organic and trusted way. As with the expert reviewers, these practitioners participated voluntarily and received no financial incentives. We compiled these metrics to assess various aspects of the model’s performance in a clinical context. Each metric was rated on a 5-point Likert scale, where 1 signifies the lowest score and 5 the highest. More details on these metrics are reported in Table 4. In this way, we evaluated MenstLLaMA with the experts’ involvement in a simulated clinical setting, providing insights that extend beyond quantitative measures. The results of this evaluation are discussed in the “Results” section.

#### User Evaluation

For a more comprehensive evaluation of the MenstLLaMA, a user study was conducted with 200 participants, comprising 181 females and 19 males, representing a diverse range of sociodemographic and professional backgrounds. Participants ranged in age from 18 to 52 years (mean 28.4 years, SD 7.3 years). Of the 200 participants, 144 (72%) identified as urban residents, 46 (23%) as semiurban, and 10 (5%) as rural. Educational backgrounds included high school education (14/200, 7%), undergraduate education (98/200, 49%), postgraduate education (62/200, 31%), and doctoral or equivalent professional qualifications (26/200, 13%). The cohort included engineering and medical students, researchers, practicing doctors, allied health professionals, and industry professionals from the technology, finance, and consulting sectors. The study was conducted primarily across India, Bangladesh, Nepal, and Bhutan, thus incorporating regional and cultural diversity into the evaluation process.

Participants were recruited through open calls circulated on university bulletin boards, professional forums, and social media, with the goal of reaching a broad user base. All participants volunteered and received no compensation, which we acknowledge may introduce an interest-based self-selection bias. Before participation, informed consent was obtained via a consent modal displayed through the ISHA web interface, where users were briefed about data usage for research purposes. No personal or identifiable information was collected. Participants were instructed to interact with MenstLLaMA through the ISHA chatbot interface and provide feedback. They evaluated the model’s responses using the same qualitative metrics (see Table 4 for the full set of metrics) employed in the simulated clinical evaluation by medical practitioners, with 2 additional metrics: *tone* and *flow*. These additional metrics were especially significant in the context of menstrual and gynecological health, where sensitivity and compassionate communication are paramount. This pilot study was designed to provide initial feedback on the system’s relevance and accessibility, rather than to reflect broader population-level adoption. Insights from this study will inform future iterations and deployment planning, including projections for user reach and usage scale.

### Ethical Considerations

This study did not require new approval from the institutional ethics committee, as it did not involve any clinical interventions, and participation was entirely voluntary and anonymous. Human participants were involved in both the expert evaluations and the user study. All participants participated voluntarily, and informed consent was obtained before participation. The user study was conducted using an interactive chatbot interface in a controlled research setting, where participants were presented with a consent notice outlining the study’s purpose, data usage, and privacy safeguards. No personally identifiable or sensitive information was collected at any stage. No compensation was provided for participation in this study.

## Results

We evaluated MenstLLaMA against the baseline LLMs using both automatic and human-based approaches.

Table 5 reports the automatic scores for all models, computed using BLEU, METEOR, ROUGE-L, and BERTScore. In our experiments, we tested various prompting strategies for the baseline LLMs: zero-shot (no example given in the prompt), one-shot (single example), and two-shot (2 examples). MenstLLaMA achieved the highest scores in BLEU (0.059) and BERTScore (0.911). Meanwhile, Claude-3 and GPT-4o achieved the highest scores for METEOR (0.321) and ROUGE-L (0.253), respectively. Interestingly, Gemini1.5-Pro and LLaMA 3 demonstrated reliable but slightly lower scores, indicating stable performance on specific tasks. Newer models such as Mistral and Orca 2 delivered strong BLEU and METEOR scores, but occasionally underperformed on ROUGE-L compared with others. Falcon, while showing some promise, consistently lagged across most metrics. In the zero-shot scenario, the closed-source models (GPT-4o, Claude-3, and Gemini1.5-Pro) outperformed all open-source models. Introducing a single example in the one-shot setting improved performance across most models. Notably, Mistral showed a significant jump in performance, achieving the highest BERTScore (0.905) and even surpassing the closed-source models in this setting. A similar trend was observed in the two-shot setting, where Mistral continued to gain over its zero-shot and two-shot performance, once again outperforming closed-source models. Overall, closed-source models consistently performed well across all scenarios, demonstrating robust generalization capabilities even in zero-shot conditions. However, with few-shot prompting, certain open-source models, such as Mistral and Phi 3, showed remarkable improvements. Mistral, in particular, delivered a competitive performance with closed-source models in both one-shot and two-shot settings. While most models benefited from additional examples, the degree of improvement was not uniform. Open-source models generally gained more from few-shot prompting compared with their closed-source counterparts. Notably, our proposed model, MenstLLaMA, exhibited strong performance across all metrics without relying on few-shot examples. This indicates that fine-tuning on the MENST dataset has effectively adapted the model to the menstrual health domain.

Based on the automatic evaluation scores, we selected the top 4 performing models for further assessment: MenstLLaMA, GPT-4o, Claude-3-opus, and Mistral. These models underwent an additional round of evaluation by clinical experts using the expert-compiled gold test set. To ensure unbiased assessment, we engaged evaluators (experts) who were not involved in curating the gold test set. MenstLLaMA consistently outperformed the other models, including the gold-standard responses, achieving mean expert ratings of 3.97 for *relevance*, 4.48 for *understandability*, 3.90 for *preciseness*, 4.00 for *correctness*, and 3.41 for *contextual sensitivity* (all out of 5). These results were supported by substantial interrater agreement (Fleiss κ≈0.68), indicating the model’s ability to generate contextually appropriate and medically reliable responses. Notably, the fact that MenstLLaMA’s outputs were frequently preferred over the gold-standard responses underscores its effectiveness in interpreting and applying domain-specific knowledge.

We subsequently carried out a second phase evaluation with medical practitioners in a simulated clinical setting using ISHA, MenstLLaMA’s chatbot interface. In this assessment, the model scored 3.5 in *relevance*, indicating that while the responses were generally pertinent, there were gaps in fully addressing specific queries. For instance, when asked about the safety of using menstrual cups during infections, the model offered a general hygiene tip but failed to mention medical contraindications. *Understandability* was rated at 3.6, reflecting that most responses were clear and easy to comprehend, although explanations involving hormonal regulation occasionally used overly technical language. *Preciseness* received a score of 3.1, suggesting that although the chatbot provides relevant information, it shows some inconsistency in maintaining concise and directly relevant answers. *Correctness* was rated at 3.5, underscoring that while the chatbot delivered accurate responses for common issues, updates are needed, particularly regarding recent guidelines (ie, the model incorrectly cited outdated WHO [World Health Organization] iron supplementation guidelines in an instance) and less frequently addressed topics. Notably, the model excelled in *context sensitivity,* scoring 4, demonstrating a strong ability to respond empathetically and understand the emotional nuances in interactions.

Finally, in the user case study, the users rated the model across 7 key metrics on a 5-point scale. Relevance received a relatively high score of 4.3, indicating that users generally found the responses pertinent to their queries. Understandability earned the highest score (4.7), reflecting the clarity and ease with which users could comprehend the information provided. Preciseness was rated 4.28, suggesting that while responses were generally concise and to the point, there remains a need to fine-tune outputs to avoid verbosity and redundancy. Correctness received a score of 4.1, affirming that users found the chatbot’s information mostly accurate, though some responses could benefit from more specific and updated details. The evaluation also highlighted positive feedback in flow (4.2) and tone (4.6), with users appreciating the polite and empathetic nature of the interaction. However, context sensitivity scored lower, at 3.9, suggesting room for improvement in how well ISHA adapts to and understands subtle emotional cues within conversations.

**Table 5.** Automatic scores of the models using the BLEU^a^, METEOR^b^, ROUGE-L^c^, and BERTScore^d^ metrics. We applied zero-shot and few-shot (1 and 2 shots) prompting techniques to the baseline models. The scores are computed with respect to the expert-compiled test set.^e^

Technique and models	BERTScore	BLEU	METEOR	ROUGE-L
Zero-shot				
GPT4o	0.896	0.052	0.256	*0.253*
Gemini1.5-Pro	0.887	0.034	0.210	0.215
Claude-3	0.888	0.054	*0.321*	0.227
LLaMA 3^f^	0.881	0.046	0.293	0.201
GPT 2	0.874	0.045	0.219	0.219
Phi 3	0.885	0.047	0.281	0.207
Orca 2	0.882	0.040	0.259	0.197
Mistral	0.888	0.043	0.301	0.201
Falcon	0.865	0.021	0.213	0.143
One-shot				
GPT4o	0.891	0.056	0.256	0.242
Gemini1.5-Pro	0.879	0.043	0.223	0.221
Claude-3	0.904	0.040	0.305	0.230
LLaMA 3	0.881	0.044	0.298	0.202
GPT 2	0.888	0.035	0.237	0.211
Phi 3	0.898	0.046	0.273	0.205
Orca 2	0.879	0.047	0.265	0.183
Mistral	0.905	0.051	0.292	0.182
Falcon	0.857	0.031	0.197	0.159
Two-shot				
GPT4o	0.892	0.056	0.260	0.246
Gemini1.5-Pro	0.878	0.045	0.224	0.220
Claude-3	0.900	0.045	0.305	0.226
LLaMA 3	0.879	0.044	0.302	0.200
GPT 2	0.886	0.032	0.236	0.215
Phi 3	0.901	0.051	0.276	0.204
Orca 2	0.875	0.042	0.265	0.179
Mistral	0.907	0.054	0.296	0.180
Falcon	0.855	0.032	0.195	0.160
Proposed model				
MenstLLaMA	*0.911*	*0.059*	0.290	0.224

a BLEU: Bilingual Evaluation Understudy.

b METEOR: Metric for Evaluation of Translation with Explicit Ordering.

c ROUGE-L: Recall-Oriented Understudy for Gisting Evaluation-Longest Common Subsequence.

d BERTScore: Bidirectional Encoder Representations from Transformers Score.

e Scores in italics indicate the highest performance in each metric.

f LLaMA: Large Language Model Meta AI.

## Discussion

### Principal Findings

In this study, we introduced the MENST dataset and MenstLLaMA. The MENST dataset was used to train the MenstLLaMA, a novel open-source LLM designed specifically for the menstrual health domain. Our comprehensive evaluation highlighted MenstLLaMA’s strong performance across both automated and expert evaluations. It outperformed several state-of-the-art general-purpose LLMs (both open and closed source) on automatic and human evaluation metrics, with users and clinicians rating its responses as effective in delivering accurate, accessible, and culturally sensitive menstrual health information.

Our domain-specific MenstLLaMA demonstrated competitive performance compared with state-of-the-art general-purpose LLMs, including open-source and closed-source LLMs, achieving superior results in automatic evaluation metrics, such as BLEU (0.059) and BERTScore (0.911), all without relying on few-shot examples. While closed-source models such as GPT-4o and Claude-3 performed robustly in zero-shot settings, MenstLLaMA’s consistent performance highlights the success of domain-specific adaptation. These findings underscore the potential of fine-tuning general-purpose LLMs for specialized domains to generate fluent, medically adequate, and contextually relevant responses. The quantitative improvements observed with MenstLLaMA are further supported by our in-depth qualitative evaluation pipeline. These qualitative gains, especially crucial in sensitive health domains, demonstrate the value and necessity of a specialized model like MenstLLaMA.

Clinical expert evaluations showed encouraging results, with MenstLLaMA’s responses often preferred over the gold-standard answers. This preference suggests that the model effectively captures domain knowledge and aligns well with clinical expertise. The model’s ability to generate responses that experts rated as more appropriate than curated answers underscores its potential as a reliable source of menstrual health information. The simulated clinical setting evaluation, conducted using the ISHA chatbot, revealed both strengths and areas for improvement. Medical practitioners interacted with ISHA by simulating patient scenarios and evaluating the model’s responses based on a set of qualitative metrics. While the model demonstrated strong empathetic capabilities (*context sensitivity*: 4.0), its performance in *preciseness* (3.1) indicates room for improvement in generating concise and focused responses. The modest score in *preciseness* reflects MenstLLaMA’s intended role as a supportive, culturally sensitive informational tool rather than a clinical decision-making system. Given the sensitivity and stigma surrounding menstrual health in India, the model prioritizes empathetic and elaborative responses to foster a safe, private environment for discussing sensitive topics while minimizing hallucinations. This approach naturally affects response length. The findings indicate that while domain-specific training successfully improved the model’s emotional intelligence, further work may be needed to enhance its ability to provide more precise information without compromising empathy or cultural appropriateness.

These findings position MenstLLaMA as a strong candidate for pilot implementation within digital support services, particularly in regional health initiatives focused on women’s health. A potential next step involves trialing an enhanced version of MenstLLaMA in collaboration with community health workers or digital health platforms in South Asia, where MHE remains both urgent and underserved.

User evaluation results were particularly promising for real-world applications. High scores in understandability (4.7) and relevance (4.3) suggest that MenstLLaMA effectively bridges the gap between clinical accuracy and user comprehension. Its strong performance in tone (4.6) and flow (4.2) further demonstrates the model’s ability to maintain engaging, empathetic conversations while delivering accurate and relevant health information.

To this end, the strength of MenstLLaMA lies not only in its technical performance but also in its capacity to deliver context-sensitive health education at scale. Its ability to provide culturally tailored, empathetic responses opens new avenues for reaching populations who may not engage with traditional MHE due to stigma or limited access. These findings suggest that specialized LLMs like MenstLLaMA can act as an accessible bridge between formal health care systems and community needs, particularly in low-resource settings. Moreover, the model’s preference over gold-standard answers in expert evaluation highlights the potential of data-driven approaches to complement and support clinician-led education strategies.

### Limitations

Our study has several limitations. While the MENST dataset covers a broad range of topics, it does not fully represent all cultural perspectives or demographics. Although diverse user groups were included in our evaluation, longitudinal studies are needed to assess the sustained impact of the intervention. The model’s current text-based interface may limit accessibility for users with varying communication needs or low digital literacy. As menstrual health norms evolve, periodic updates will be necessary to maintain relevance. As with all LLMs, MenstLLaMA is prone to hallucinations, producing fluent but incorrect outputs, which can pose risks in health-related contexts. Although we implemented safeguards through prompt design and fine-tuning, these cannot entirely eliminate such risks. There is also potential for ethical misuse if the model is deployed without appropriate oversight, emphasizing the importance of human-in-the-loop supervision and responsible implementation. Additionally, the model may reflect inadvertent biases, stemming from both the training data and the background of evaluators. As participation was voluntary and likely attracted individuals already interested in technology or menstrual health, selection bias may have influenced the user evaluation outcomes. While our model has shown promise in controlled settings, scaling it for real-world use presents several challenges, such as the need to expand demographic representation, enhance context sensitivity, and integrate the model with broader health care interventions. Additionally, real-world deployment must address practical concerns such as system integration, digital literacy gaps, user trust, and data privacy across diverse user groups.

### Conclusions

MenstLLaMA represents a significant advancement in the application of AI to MHE. Its performance across multiple evaluation metrics demonstrates that domain-specific language models can effectively bridge the gap between clinical accuracy and accessible health communication. The model’s strong results in both technical metrics and user evaluations suggest its potential as a valuable tool for addressing the critical need for accurate, empathetic, and culturally sensitive menstrual health information. While there is room for improvement, particularly in areas such as precision and context sensitivity, the overall findings indicate that MenstLLaMA successfully meets its primary goal of making MHE more accessible and understandable. As we continue to refine and expand this approach, specialized language models like MenstLLaMA could play an increasingly important role in improving access to quality menstrual health information and in reducing stigma on a global scale. Future work will focus on exploring responsible deployment pathways, including partnerships with health care organizations to pilot MenstLLaMA in targeted regional settings.

## Abbreviations

AI: artificial intelligence
BERTScore: Bidirectional Encoder Representations from Transformers Score
BLEU: Bilingual Evaluation Understudy
FAQ: frequently asked question
ISHA: Intelligent System for Menstrual Health Assistance
LLaMA-3: Large Language Model Meta AI 3
LLM: large language model
METEOR: Metric for Evaluation of Translation with Explicit Ordering
MHE: menstrual health education
PEFT: parameter-efficient fine-tuning
QA: question-answer
ROUGE-L: Recall-Oriented Understudy for Gisting Evaluation-Longest Common Subsequence
